# Cancer cell‐intrinsic type 1 interferon: production, signaling, and outcomes within sex‐biased, female malignancies

**DOI:** 10.1002/1878-0261.70311

**Published:** 2026-07-28

**Authors:** Ashlyn Conant, Nora Badiner, Sharon Asariah, Kiera McGivney, Vishwa Shah, Tise Suzuki, Yevgeniya J. Ioffe, Juli J. Unternaehrer

**Affiliations:** ^1^ Division of Biochemistry, Department of Basic Sciences Loma Linda University CA USA; ^2^ Division of Gynecologic Oncology, Department of Gynecology and Obstetrics Loma Linda University CA USA; ^3^ Department of Biochemistry Whitworth University Country Homes WA USA; ^4^ Present address: Division of Oncology, Department of Gynecologic Oncology Saint Luke's Hospital Kansas City MO USA; ^5^ Present address: School of Medicine California University of Science and Medicine Colton CA USA; ^6^ Present address: Department of Biology/Allied Health Southern Adventist University Collegedale TN USA

**Keywords:** breast cancer, DNA damage, innate immunity, interferon, ovarian cancer, sex‐bias, signaling, STAT1, STING

## Abstract

Type 1 interferons (IFN‐1), such as IFN‐α and IFN‐β, are the most well‐studied interferon class due to their abundant expression, widespread signaling, and potent immunomodulatory functions. Although the role of these cytokines in mediating antiviral defense responses and immune activation has been well‐studied, recent studies have suggested they play a more nuanced role in directing cell‐autonomous responses in cancer. The role of cancer cell‐autonomous IFN‐1 production and signaling is complex and context dependent. IFN‐1 has been shown to participate in both tumor‐suppressing and ‐promoting activities based on the intensity and duration of the IFN‐1 signal received. The cell‐intrinsic roles appear to be immune‐independent, mediating cancer cell responses to traditional therapies and regulating tumor progression. This review aimed to summarize the various roles of IFN‐1 in cancer, with a focus on female‐related malignancies—including ovarian, breast, cervical, and endometrial cancers—emphasizing the molecular mechanisms surrounding IFN‐1 production and signaling, downstream functional outcomes, and therapeutic implications essential to understanding the pro‐ and antitumor roles of IFN‐1.

## Graphical abstract

The cell‐autonomous roles of interferon type 1 vary based on duration and intensity. While acute, robust signaling results in cancer cell cytotoxic effects, sustained, low‐level signaling is associated with tumor‐promoting effects. This review summarizes current research of IFN‐1 in cancer and within the clinical setting, with an emphasis on female‐associated malignancies. Created in biorender. Conant, A. (2026) https://BioRender.com/32tp3uo.

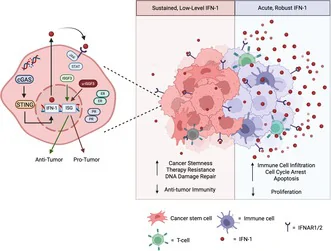

**Abbreviations**
ARF: ADP‐ribosylation factor
AZA: 5‐azacytidine
BAG2: Bcl2‐associated athanogene 2
BRCA: BReast CAncer gene
CAF: cancer associated fibroblast
CDK: cyclin‐dependent kinase
cGAMP: cyclic guanosine monophosphate‐adenosine monophosphate
cGAS/STING: cyclic GMP‐AMP synthase/stimulator of interferon genes
CT: chemotherapy
DAMP: damage‐associated molecular patterns
DDR: DNA damage repair
EMT: epithelial‐mesenchymal transition
ER: estrogen receptor
FDA: Federal drug administration
FRT: female reproductive tract
GAS: gamma‐activated sequence
HDAC: histone deacetylase
HDACi: histone deacetylase inhibitors
HGSOC: high‐grade serous ovarian cancer
HSV2: Herpes simplex virus 2
IFI6: interferon alpha inducible protein 6
IFIT1/3: interferon‐induced protein with tetratricopeptide repeats 1/3
IFITM1/2: interferon‐induced transmembrane protein 1/2
IFN‐1: Type 1 Interferon
IFNAR: interferon alpha receptor
IRDS: IFN‐related DNA damage resistance signature
IRF: interferon regulatory factor
IRS: insulin receptor substrate
ISG: interferon‐stimulated gene
ISGF3: interferon‐stimulated gene factor 3
ISRE: interferon‐stimulated response elements
IκBα: inhibitor of kappa B α
JAK1: Janus kinase 1
MAPK: mitogen‐activated protein kinase
MnCl2: manganese chloride
MSI‐H: microsatellite‐high
MTD: maximum tolerated dose
NF‐κB: nuclear factor kappa‐light‐chain‐enhancer of activated B cells
NIS: sodium iodide symporter
NLRX1: NOD‐like receptor X1
PAMP: pathogen associated molecular pattern
PD‐L1: programmed death ligand 1
PFS: progression free survival
PI3K/AKT: phosphoinositide‐3‐kinase/protein kinase B
PR: progesterone receptor
PRR: pattern recognition receptors
PTEN: phosphatase and tensin homolog
PV: polycythemia vera
RIG‐1: retinoic acid‐inducible gene I
RT: radiation therapy
SLP: synthetic long peptide vaccine
SOCS1/3: suppressor of cytokine signaling 1/3
STAT: signal transducer and activator of transcription
STIC: serous tubal intraepithelial carcinoma
STING: stimulator of interferon genes
TLR: Toll‐like receptor
TRIM29: tripartite motif‐containing 29
TYK: tyrosine kinase
u‐ISGF3: unphosphorylated interferon‐stimulated gene factor 3
USP18: ubiquitin‐specific peptidase 18

## 1 Introduction

**Fig. 1:**
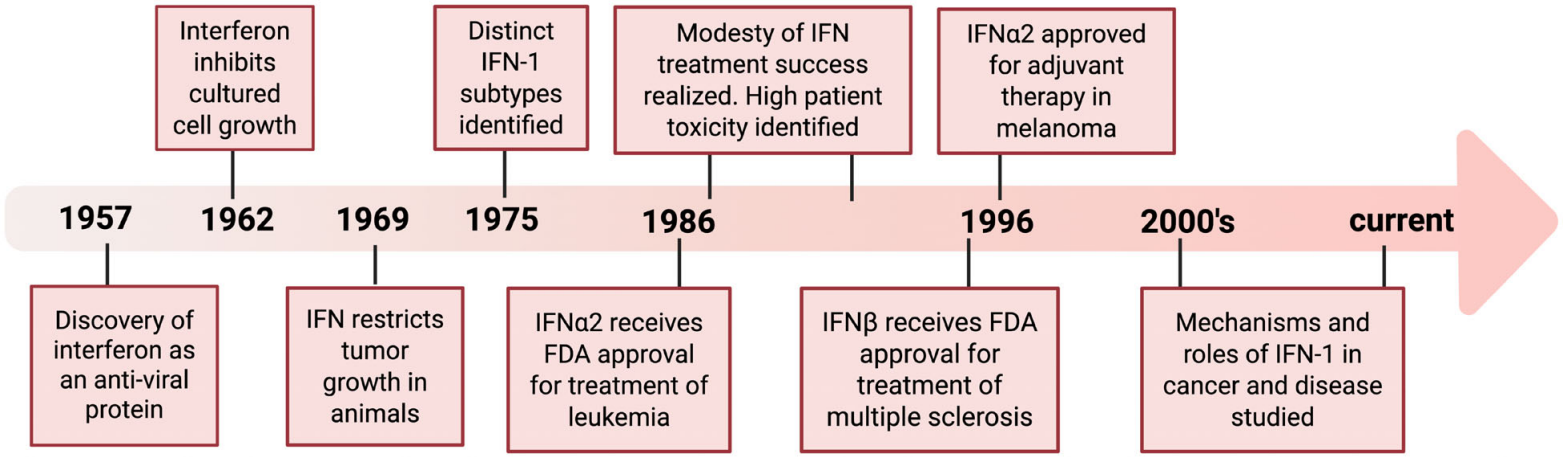
Brief history of major milestones in the discovery and use of interferon (IFN). IFN was identified as a potent antiviral protein, which led to research efforts focused on elucidating the roles of IFN in cell culture and mouse models. IFN was found to inhibit tumor growth in mice, heavily contributing to enthusiasm over its potential as a cancer therapeutic. IFN‐α received FDA approval for the treatment of leukemia and other disease types, followed by IFN‐β. However, success was limited, as IFN was shown to only be effective in a small number of cancers. High patient toxicity was quickly identified, which limited the use of this drug. More recent efforts have focused on identifying the precise pro‐ and antitumor roles of these molecules. Created in biorender. Conant, A. (2026) https://BioRender.com/rqgsnxu.

IFNs first attracted attention as a potential cancer therapeutic in the 1960s, with Ion Gresser being among the first scientists to publish on the use of IFN‐1 to treat virally induced cancer. These molecules were found to have powerful antitumor abilities, but the full extent of their action, both in cancer and in physiologic immune settings, was poorly defined at the time. Gresser's groundbreaking 1969 study excited medical, scientific, and public health communities with the notion that IFNs may play a role in fighting tumorigenesis (Fig. 1) [1].

Almost 20 years later, IFN‐α became the first Federal Drug Administration (FDA)‐approved immunotherapeutic agent. A plethora of initial studies in many clinical settings reflected the early enthusiasm for the potential of IFN as a therapeutic. Success was seen in some patients; however, both dose and duration of treatment contributed to high rates of toxicity, ultimately limiting further clinical development and therapeutic use [2]. While many studies and clinical trials have now established that exogenous IFN‐1 is not a standalone cancer therapy, ongoing studies continue to reveal its nuanced roles in both immune and cancer cells. Although originally heralded as the future of cancer treatment, a much more complex paradigm regarding the functions of IFNs has emerged. IFNs were once thought to act in an antitumor manner exclusively via immunomodulation; however, a growing body of evidence suggests that these molecules may also act directly on tumor cells, independent of the immune system, to influence tumor development and progression.

Cancer cell‐autonomous IFN‐1 production and signaling, defined as IFN‐1 that is initiated and acts within malignant epithelial cells independent of exogenous IFN derived from immune or stromal compartments, has been observed to influence tumor cell fate, altering cancer cell phenotypes and directing responses to treatment. These effects can result in both promotion and impediment of disease progression, depending on specific cellular and molecular contexts. While IFN‐1 signaling has historically been studied primarily through its immunomodulatory functions within the tumor microenvironment, emerging evidence suggests that tumor‐intrinsic IFN‐1 signaling may represent a critical and understudied regulator of tumor biology. Outside of oncology, intrinsic IFN‐1 signaling has been implicated in fundamental cellular processes including aging, stem cell maintenance, and the induction of senescence, highlighting its capacity to influence cell states and phenotypes [3]. However, despite these defined roles in other biologic systems, the cell‐intrinsic roles of IFN‐1 in cancer remain poorly understood. Current evidence suggests a functional duality of IFN‐1 within cancer, emphasizing that the consequences of these signals are highly dependent on disease context. Therefore, a more comprehensive understanding of the mechanisms and roles of cancer cell‐intrinsic IFN‐1 signaling must be obtained to successfully target these pathways for disease treatment.

Sex as a biologic variable is fundamental, yet understudied and underintegrated in cancer biology. IFN‐1 signaling and production pathways exhibit well‐established sexual dimorphism, shaped by X‐chromosome gene dosage, incomplete X‐inactivation of immune regulatory factors, and marked transcriptional sensitivity to steroid hormones [4]. Female tissues specifically demonstrate pathways, such as estrogen and progesterone receptor signaling, which intersect directly with canonical IFN‐1 signaling pathways to modulate IFN‐1 responsiveness. IFN‐1 signaling regulatory features are frequently rewired in female‐related malignancies such as ovarian, breast, endometrial, and cervical cancers, where chronic inflammatory signaling, DNA‐damage response, and immune modulation are central to disease biology. While the traditional paradigm of IFN‐1 has been established as a sustained tumoricidal and cytotoxic pathway, emerging data compel reevaluation of this framework.

In this review, we focus on IFN‐1 in female‐specific malignancies, providing a unique summary of how anatomical and sex‐biased pathway regulation intersects with therapeutic stress and hormonal signaling to reveal principles that might otherwise be obscured in pan‐cancer analyses.

## 2 IFN‐1 production

IFN‐1 are pleiotropic cytokines involved in innate immunity [5], classically known for their roles in viral defense and immune activation [6, 7, 8]. The IFN‐1 family consists of 17 subtypes, all sharing about 70% homology; 13 IFN‐alpha (IFN‐α) members, IFN‐beta (IFN‐β), IFN‐epsilon (IFN‐ε), IFN‐kappa (IFN‐κ), and IFN‐omega (IFN‐ω) [9, 10]. IFN‐α/β are the most widely expressed, the most well‐characterized, and perhaps the most biologically important IFNs. IFN‐κ is almost exclusively produced by keratinocytes, while IFN‐ω is produced predominately by leukocytes (Table 1).

**Table 1:** List of IFN‐1 subtypes, genetic members, primary cellular sources, and notable physiologic characteristics or functions.

Cytokine subtype	Member (genes)	Cellular sources	Notes
IFN‐α (alpha)	IFNA1, IFNA2, IFNA4, IFNA5, IFNA6, IFNA7, IFNA8, IFNA10, IFNA13, IFNA14, IFNA16, IFNA17, IFNA21	Plasmacytoid dendritic cells, monocytes, macrophages	Highly anti‐viral, most diverse subtype
IFN‐β (beta)	IFNB1	Most cell types	Viral/immunomodulatory roles
IFN‐ε (epsilon)	IFNE	Mucosal epithelial cells	Provides mucosal immunity, protects against sexually transmitted diseases
IFN‐κ (kappa)	IFNK	Keratinocytes	Key for skin barrier defense
IFN‐ω (omega)	IFNW1	Leukocytes, monocytes, dendritic cells	Similar to alpha, less diverse subtypes, less abundant

### 2.1 Classic IFN‐1 induction

During infection, IFN‐1 production is initiated upon detection of extracellular or cytosolic nucleic acids, or non‐nucleic acid pathogen associated molecular patterns (PAMPs) by pattern recognition receptors (PRRs) [11, 12, 13, 14, 15]. The expression of PRRs is cell type–restricted: endosomal Toll‐like receptors (TLR) are predominantly expressed in specific immune cells (e.g., plasmacytoid dendritic cells and B‐cells) [16], whereas cytosolic RNA sensors [14] (RIG‐I (retinoic acid‐inducible gene I) [17, 18, 19]), and the DNA‐sensing cGAS–STING (GMP‐AMP synthase/stimulator of interferon genes) [20, 21] axis are broadly expressed among many epithelial and stromal cell types. These proteins are the main organizers and central framework governing context specific IFN‐1 transcriptional activation [22].

IFN‐1 molecules are classically produced in an acute and robust manner, wherein potent, large amounts of IFN are quickly made and secreted from a cell, binding surrounding or host cells, signaling in both a paracrine and autocrine manner. The half‐life of circulating IFN protein is relatively short and subtype specific, spanning from 30 min to a few hours, consistent with the concept that a key characteristic of this signaling cascade is the transient nature of the ligand [23, 24, 25]. However, recent studies are beginning to characterize the complex nature of IFN production, revealing that IFN‐stimulated transcription and response depend on both the duration and magnitude of the IFN‐1 signal.

IFN‐1 production is tightly regulated by several negative regulatory mechanisms that function at the transcriptional, signaling, and receptor levels to prevent excessive inflammation and signaling pathway activation. At the transcriptional level, TRIM29 (tripartite Motif‐Containing 29), NLRX1 (NOD‐like receptor X1), and various other ubiquitin‐editing enzymes block upstream signaling by targeting adaptor proteins, such as STING, thus blocking IFN‐1 production initiation and response [26, 27]. Following activation of IFN‐1 cytokines, feedback inhibitors such as SOCS1 and SOCS3 (suppressor of cytokine signaling 1/3) are activated and tightly regulate downstream IFN‐1 signaling by inhibiting JAK kinase activity [28]. Ubiquitin‐Specific Peptidase 18 (USP18) competes with janus kinase 1 (JAK1) to bind to interferon alpha receptor 2 (IFNAR2) and signal transducer and activator of transcription 2 (STAT2) to restrict downstream signal propagation [29, 30]. Receptor internalization and degradation further limit the duration of IFN‐1 signal availability [31]. Together, these mechanisms enforce the transient nature of the IFN‐1 response, ensuring that antiviral action is acute and does not extend into a chronic inflammatory or autoimmune program. Reviews by both Ivashkiv and Donlin, and McNab et al., provide exhaustive expansions on the classic mechanisms contributing to IFN‐1 production [32, 33].

### 2.2 IFN‐ε as a distinct subtype

Interestingly, and highly relevant to the discussion on IFN‐1 related production within female‐associated pathologies, IFN‐ε is an IFN‐1 that is constitutively expressed by epithelial cells within the female reproductive tract (FRT) [34]. While IFN‐ε is best characterized in the FRT, it is also expressed in other mucosal tissues, including the lung and gastrointestinal epithelium, as well as in the male reproductive tract [35, 36]. Unlike other IFNs, IFN‐ε is not primarily regulated by pathogen response receptors but is instead hormonally regulated by both estrogen (which increases expression) and progesterone (which decreases expression) with distinct transcriptional activation, supporting locally constitutive and homeostatic expression that does not invoke an immune response [37, 38, 39].

In contrast to other IFNs, which are robustly induced in response to infection or pathogenic exposure via PRRs, IFN‐ε is regulated in concurrence with distinct stages of pregnancy or with phases of the menstrual cycle, marked by hormonal fluctuation [40, 41]. IFN‐ε's unique expression has been shown to play a key role in the regulation of spatially restricted innate immunity, protecting the FRT from viral and bacterial infections, such as herpes simplex virus 2 (HSV2) and *Chlamydia muridarum*, via promotion of interferon‐stimulated gene (ISG) programs that fortify the epithelial barrier integrity while avoiding inflammatory activation that would otherwise compromise reproductive homeostasis [39, 42, 43, 44].

In the context of female‐associated pathologies, IFN‐ε participates in canonical IFN‐1 signaling [45, 46]. In both ovarian cancer and pancreatic cancers, IFN‐ε has been found to be highly downregulated, consistent with a potential tumor‐suppressive role. Loss of IFN‐ε after menopause [39] may contribute to the increased rates of ovarian cancer development in postmenopausal women. Marks et al. demonstrated that exogenous IFN‐ε decreases ovarian cancer cell proliferation and promotes apoptosis, while reducing tumor burden and peritoneal metastases in tumor‐bearing mouse models [47]. This tumor‐suppressive role has been validated in pancreatic cancer, as well, as Barriga et al. found that IFN‐ε deletion in pancreatic cancer was associated with tumor dissemination and immune evasion [48]. In cervical cancer, IFN‐ε has been reported to be upregulated in HPV‐positive cases, suggesting a complex regulatory role in mucosal immunity and antiviral defense within specific tissues [49]. In the context of pregnancy, IFN‐ε expression in the cervix, myometrium, and chorioamniotic membranes has been linked to immune protection and pregnancy outcomes, with high expression associated with preterm birth, further indicating its role in protecting the female reproductive tract and gestation [41].

These studies suggest that IFN‐ε contributes to hormonally regulated, tissue‐specific modulation of IFN‐1 responses, particularly within the FRT. However, IFN‐ε represents a unique example of an IFN whose regulation and function are strongly directed by tissue context and hormonal states, offering insight into sex‐biased immune regulation and highlighting the need for further research into its role across both physiological and pathological, female and male settings.

### 2.3 IFN‐1 induction in cancer

**Fig. 2:**
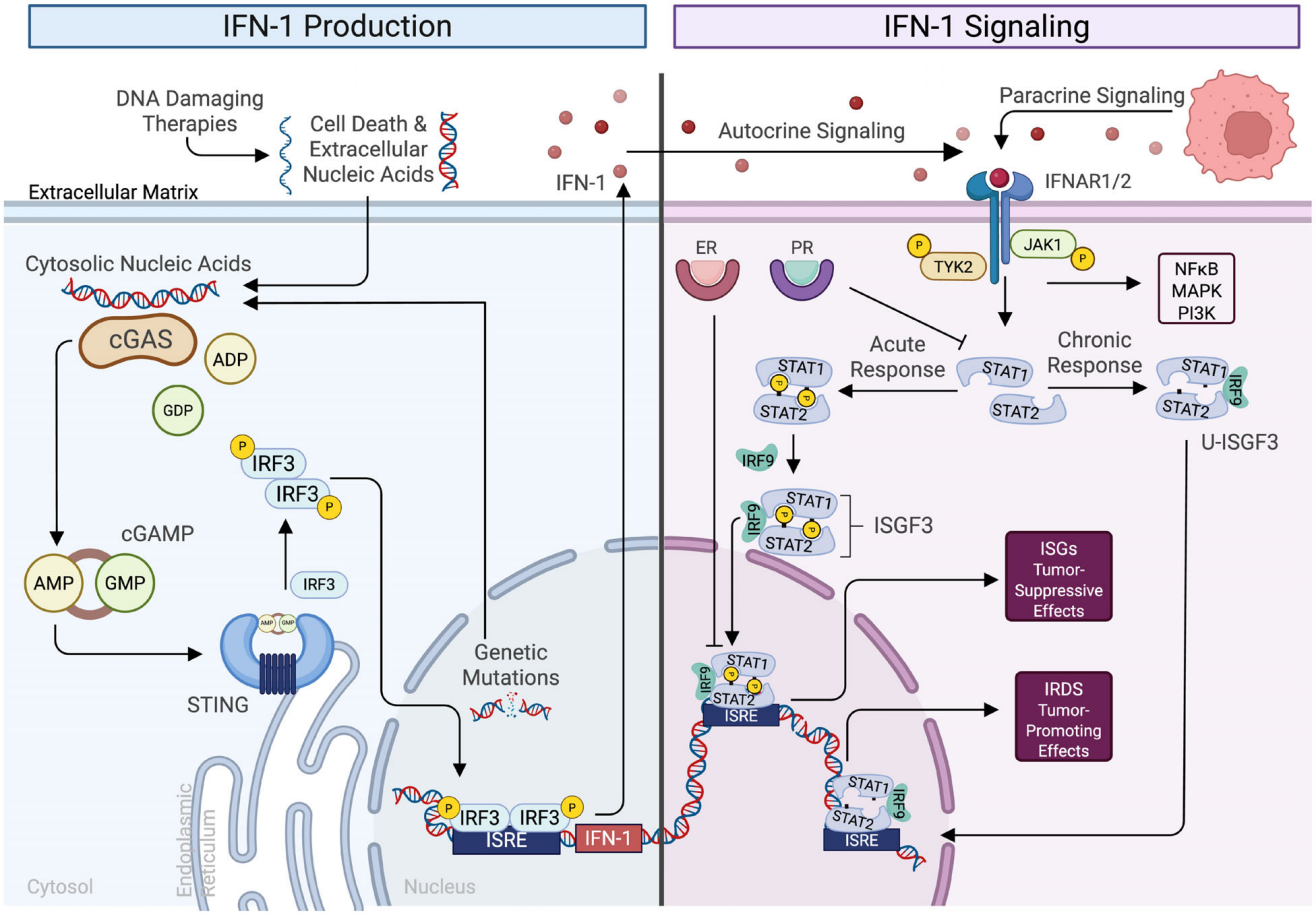
Cancer cell intrinsic interferon type 1 (IFN‐1) production and response. Upon DNA damaging therapy‐induced release of nucleic acids, extracellular or cytosolic nucleic acids are detected by cyclic GMP‐AMP synthase (cGAS) which catalyzes the synthesis of cyclic guanosine monophosphate‐adenosine monophosphate (cGAMP), which binds to and activates stimulator of interferon genes (STING). Activated STING leads to the phosphorylation of interferon regulatory factor (IRF) 3, allowing for translocation into the nucleus, binding to interferon‐stimulated response elements (ISREs), and inducing gene expression of IFN‐1. High rates of genetic mutations and the presence of intracellular programmed death ligand‐1 (PD‐L1) also activate the cGAS/STING pathway. Secreted IFN acts in both paracrine and autocrine fashion. IFN‐1 binds to interferon alpha and beta receptor 1/2 (IFNAR1/2) and activates Janus kinase 1/signal transducer and activator of transcription (JAK/STAT) signaling. An acute IFN‐1 response triggers the canonical response, with phosphorylation of STAT1 via JAK1, followed by the binding of IRF9, forming the interferon stimulated gene factor 3 (ISGF3) complex which translocates into the nucleus and binds to insulin‐stimulated response elements (ISREs), activating expression of tumor‐suppressive ISGs. A chronic IFN‐1 response results in the formation of the unphosphorylated ISGF3 complex (u‐ISGF3), which activates expression of genes that promote a protumor phenotype, including the interferon‐related DNA damage signature (IRDS). IFN‐1 can also activate noncanonical signaling pathways such as nuclear factor kappy‐light‐chain‐enhancer of activated B cells (NFκB), mitogen‐activated protein kinase (MAPK), and phosphoinositide‐3 kinase (PI3K). Intracellular estrogen receptor (ER) inhibits the ISGF3 complex from binding to ISREs, and progesterone receptor (PR) prevents the formation of ISGF3 by binding to and inhibiting STAT1 phosphorylation and binding of IRF9. Created in biorender. Conant, A. (2026) https://BioRender.com/iwmv0bx.

IFN‐1 can be produced by cancer cells, activating cell‐intrinsic signaling and activity, in response to various factors such as DNA damage resulting from chemo‐ and radiation therapies as well as genetic mutations and chromosomal instability (Fig. 2).

Production of IFN‐1 can be driven by the presence of free, extracellular, or cytoplasmic nucleic acids, resulting in the activation of both paracrine and autocrine IFN‐1 signaling that contributes to induction of cell cycle arrest and is associated with DNA damage repair program activation [3]. The common standard of care for multiple cancers includes radiation therapy (RT) or chemotherapy (CT), both of which result in widespread DNA damage and cell death. The result of these therapies includes induction of apoptosis and, in some contexts, necrosis, which can alter the extracellular environment. Depending on the mode and extent of cell death, these can lead to the release of damage‐associated molecular patterns (DAMPs) into the tumor microenvironment and occasionally systemic circulation, contributing to local inflammation and immune activation. Damaged DNA resulting from cytotoxic therapies is detected by cells close to the affected cells, resulting in the production of IFN‐1 molecules [14, 50, 51]. This IFN‐1 production appears dependent on the canonical IFN‐1 production pathway, relying on cGAS/STING to promote transcription of IFN‐1 genes [52, 53]. Studies have observed and characterized that binding of cGAS/STING to DNA occurs in a sequence‐nonspecific manner, leading to virus‐independent, robust IFN production, helping explain how this sensor can drive such a potent IFN response in cancer [54, 55]. This modality of activation, relating to the effects of CT or RT, has been seen in many cancerous and precancerous lesions in female‐related contexts including, but not limited to breast cancer, endometriosis, and ovarian cancer [56, 57, 58]. These studies defining the nonspecificity of nucleic acid activation of the cGAS/STING pathway aid in a greater understanding of how this IFN‐1 production mechanism might be affected by standard therapies and promote immune‐independent, cancer cell‐autonomous IFN‐1 production.

Several studies have found that cancer cells mount an IFN‐1 response to DNA damaging therapies [59]. Erdal and colleagues demonstrated that the treatment of several breast cancer cell lines with DNA damaging agents, such as RT or CT, increased levels of cytosolic DNA over time. This accumulation of cytosolic nucleic acids was associated with the activation of an innate immune response, by which STAT1 was phosphorylated and IFN‐1 stimulated genes were expressed at high levels. The DNA damage‐related IFN‐1 activation was dependent on the canonical cGAS/STING pathway [60]. Further defining the mechanisms and timing of IFN‐1 production with traditional treatments, one study showed that, in a mouse model, IFN‐1 production occurs concurrently with, and relies on, the presence of autophagic cells following CT treatment [61]. Another demonstrated that IFN‐1 molecules were produced by breast cancer cells between 1 to 4 days after CT, coinciding with the accumulation of massive cell death, as well as myeloid and autophagic cell infiltration [62]. These studies suggest that cancer cell‐autonomous IFN‐1 production may be universal following CT, possibly indicating a role of IFN‐1 in the action of the therapy itself. A landmark study by Sistigu et al. [62] showed that IFN‐1 activation and response, termed ‘viral mimicry’, appears necessary following CT and is predictive of successful anthracycline therapy in breast cancer [62]; similarly, a study by Burnette et al. [50] demonstrated that the efficacy of RT relies upon the induction of IFN‐1.

The precise role of IFN‐1 production by cancer cells, in response to therapy, remains nuanced. As examined in greater detail in subsequent sections, studies have suggested that IFN‐1 production and responses are closely related to traits such as therapy resistance, cancer aggression, and stemness [57, 63, 64]. Thus, the results of IFN‐1 signaling in cancer may be pro‐tumor, in contrast to the antitumor pathways introduced above. While looking upstream at IFN‐1 activators, in an ovarian cancer model, Pavan et al. [65] found that a potent IFN‐β activator, IRF1 (interferon regulatory factor), is induced following cisplatin treatment. However, rather than aiding in the response to chemotherapy, IRF1 seems to play a role in inhibiting the effectiveness of treatment, likely by preventing proliferation, which is necessary for the function of platinum‐based drugs. Of note, PD‐L1 has been found to be regulated by IRF1 [66], and has been found to participate in JAK1/STAT1 dependent signaling and regulation of proliferation in ovarian cancer cells [67, 68]. These studies provide great insight into the complicated, dualistic nature of IFN‐1 production in response to cytotoxic therapies, while highlighting the need for more complex studies to determine the exact mechanism of this phenomenon in various cancers.

IFN‐1 production has also been found to result from various genetic mutations. Alterations in DNA damage repair pathways, specifically, allow for accumulation of ss‐ and dsDNA breaks, resulting in DNA fragments, which can be released into the cytoplasm and detected by the canonical IFN‐1 production, cGAS/STING pathway [59]. One 2014 study showed that in breast cancer, co‐inactivation of both ADP‐ribosylation factor (ARF) and p53, proteins involved in response to DNA damage and cell cycle regulation, promotes oncogenic IFN‐β induction, increasing breast cancer tumorigenicity and proliferation [69]. A more recent study observed that an IFN‐1 signature can be identified in early serous tubal intraepithelial carcinoma (STIC) lesions, the presumed cells of origin for ovarian cancer, as early as p53 signature detection [70]. These studies provide evidence that cancer cells can be primed for IFN‐1 response by various genetic mutations and defective DDR proteins, suggesting that IFN might also play a role in intrinsic therapy resistance, among other phenotypes.

Phosphatase and tensin homolog (PTEN) has been found to be highly mutated in uterine, cervical, breast, and ovarian cancers. It functions to regulate several pathways, including the phosphoinositide‐3‐kinase/protein kinase B (PI3K/AKT) pathway to regulate cell proliferation, metabolism, survival, polarity, migration, and angiogenesis [71]. The nuclear import of IRF3, a protein heavily involved in the transcription of IFN‐1 molecules, is also promoted by PTEN via dephosphorylation, indicating another potential role of common genetic aberrations in IFN‐1 production in female‐related cancers [72].

### 2.4 Sex‐biased regulation of IFN‐1 production

IFN‐1 production pathways demonstrate marked sexual dimorphism, with XX‐individuals generally demonstrating heightened innate immune responsiveness compared to those with a Y chromosome [73]. Differences in these responses are partially attributed to X‐chromosome gene dosage effects, incomplete X‐inactivation of immune regulatory factors, and unique transcriptional regulation by steroid hormones. These mechanisms have been found to contribute to nucleic acid sensing, cytokine production, and downstream interferon responses, suggesting that female‐associated malignancies may arise within a unique IFN‐1 biologic context.

Several innate immune regulators involved in IFN‐1 production are encoded on the X‐chromosome, including TLR7 and 8, among other immune‐associated genes [74]. Of note, many of these loci can partially escape X‐inactivation and drive increased gene expression within XX‐individuals. Enhanced gene dosage has been associated with stronger antiviral and inflammatory IFN‐1 responses and has been implicated in female‐biased autoimmune diseases specified by chronic IFN‐1 activation [4]. While these mechanisms remain incompletely understood in the context of malignancy, they raise the possibility that other female‐associated pathologies may possess unique sensitivities to IFN‐1 pathway activation.

Steroid hormones may further dictate IFN‐1 production through direct and indirect regulation of PRRs and other nucleic acid sensing components, although many of these studies remain highly correlative. Estrogen has been found to modulate TLR7 and TLR9‐mediated IFN‐α and IFN‐1 associated IFN‐γ within plasmacytoid dendritic cells and breast cancer respectively, indicating a role in IFN‐1 activation [75, 76]. However, progesterone has been generally associated with suppression of inflammatory signaling, specifically in plasmacytoid dendritic cells, complicating the understanding of hormonal regulation of the IFN‐1 class [77]. While steroid hormone regulation of IFN‐1 production remains an understudied topic, it is likely that disruption of hormone regulation in sensitive tissues such as breast, ovarian, and endometrium may underlie tumorigenesis and altered IFN‐1 production dynamics that drive various phenotypes.

Female‐associated malignancies, particularly breast and ovarian cancers, often harbor homologous recombination deficiencies, such as BReast CAncer gene 1 or 2 (BRCA1 and BRCA2) mutations. Accumulation of cytosolic DNA resulting from replication stress, chromosomal instability, and defective DNA repair can activate cGAS‐STING signaling and promote chronic IFN‐1 production [78]. BRCA1 loss has also been found to promote the overexpression of cytosolic DNA‐sensing proteins, such as STING and interferon alpha inducible protein 6 (IFI6), as well as both IFN‐α and IFN‐β themselves, in ovarian cancer cells, suggesting a mechanism for involvement of female‐associated defective DNA damage repair (DDR) mechanisms in chronic IFN‐1 production [79]. Another study found that BRCA1 haploinsufficiency is associated with a less differentiated state and downregulation of multiple ISGs, suggesting reduced BRCA1 dosage may dampen basal IFN‐1 signaling programs that can become more apparent and functionally relevant after complete BRCA loss [80]. Together, these studies suggest that BRCA1 deficiency can drive a context‐dependent, dose‐sensitive regulation of IFN‐1, in which haploinsufficiency may decrease basal ISG expression while complete loss drives chronic activation of DNA damage associated IFN‐1 responses within female‐associated malignancies.

Further, two other studies have associated BRCA2 loss with an increase in IFN‐1 gene responses. BRCA2 loss led to an accumulation of basal DNA damage, which promoted cGAS/STING activation and drove an increase in both STAT1 and IRF3, as well as several ISG and IRDS members downstream of STAT signaling. This indicates that BRCA2 loss plays a role in both cell‐intrinsic IFN‐1 production and functional signaling, potentially driving phenotypic changes associated with BRCAness [81, 82].

## 3 IFN‐1 signaling

### 3.1 Canonical IFN‐1 signaling

Canonical IFN‐1 signaling operates through the heterodimeric IFNAR1/2. Ligand engagement induces conformational changes that allow trans‐phosphorylation and activation of JAK1, associated with IFNAR2, and tyrosine kinase 2 (TYK2) associated with IFNAR1 [83]. Activated JAK1 and TYK2 phosphorylate specific tyrosine residues on the cytoplasmic tails of IFNAR and create docking sites for STAT proteins. Although STAT1 and STAT2 molecules are the principal mediators of canonical signaling, IFN‐1 can also activate additional STAT members (STAT3, STAT4, STAT5, and STAT6) depending on activation context, signal duration, and cell type [84]. Heterodimerization of STAT1 and STAT2 is dependent on phosphorylation of the protein, with multiple phosphorylation sites on various tyrosine and serine residues. Within the canonical pathway, tyrosine phosphorylation of STAT1 is required for DNA binding of the homodimer, while serine phosphorylation of STAT1/2 modulates transcriptional potency and DNA sequence selectivity [85, 86]. Dimerized STAT1 and STAT2 associate with IRF9 to form the interferon stimulated gene factor 3 (ISGF3) [87], which translocates into the nucleus and binds ISREs to activate a wide variety of genes based on the IFN signal received [88]. Of note, STAT1 molecules can also form homodimers that bind gamma‐activated sequence (GAS) elements, while STAT3 and other isoforms contribute to various other alternative transcriptional programs that influence a variety of phenotypes. These canonical STAT‐dependent pathways indicate flexibility within the signal transduction cascade, promoting a flexible signaling mechanism that is influenced by cell type specificity, receptor density, ligand concentration, and signal intensity/duration.

### 3.2 Non‐canonical IFN‐1 signaling

IFN‐1 also activates various other noncanonical pathways such as PI3K/AKT, mitogen‐activated protein kinase (MAPK), and nuclear factor kappa‐light‐chain‐enhancer of activated B cells (NF‐κB) through the common IFNAR1/2 dimer. While not specifically described in female‐related malignancies, foundational publications aiding in the discovery and characterization of noncanonical IFN‐1 signaling are briefly summarized below. Comprehensive reviews of these pathways are available; see Cheon et al. [89] and Platanias and colleagues [90, 91].

#### 3.2.1 PI3K/AKT

Uddin and associates have published about several alternative signaling pathways activated by IFN‐1 molecules. The group found that treating human myeloma cells with IFN‐1 stimulated phosphorylation and activation of the insulin receptor substrate 1 (IRS‐1) [92], which promotes activation of PI3K and related signaling cascades [93]. Another study showed that IRS‐2 is activated by IFN‐1 and acts in a similar manner to promote PI3K signaling [94]. Cengel and Freund [95] further defined this mechanism by observing that differential IRS‐1 phosphorylation may decrease activation of the PI3K pathway and instead activate JAK1 activity and contribute to JAK/STAT responses. Ultimately, the PI3K/AKT signaling pathway is involved with a wide variety of cellular responses, promoting proliferation, growth, survival, migration, and various cancer‐specific phenotypes independent [96] or dependent on classic ISG activation [97]. It is important to note, however, that this pathway remains dispensable for the antiviral effects of IFN and related signaling, indicating its noncanonical action.

#### 3.2.2 MAPK

Another article by Uddin et al. [98] demonstrated that IFN‐1 molecules induce the rapid phosphorylation of MAPK/p38 in order to drive an IFN response. The precise role of IFN‐driven MAPK signaling has been defined using a potent p38 inhibitor, SB203580 [99]. Cells treated with this inhibitor, followed by IFN‐β, display a decrease in IFN‐1 dependent activation of ISRE and GAS containing ISGs [100]. This MAPK‐driven regulation of ISGs appears to be due to a decrease in the formation of ISGF3 and regulation of phosphorylation of STAT1 molecules. These and other studies indicate that MAPK does not exert direct kinase activity on STAT proteins [101], indicating that its contribution to ISG transcription occurs through indirect mechanisms. Rather than acting on STATs themselves, MAPK is thought to modulate transcriptional activity by influencing other co‐factors, chromatin accessibility, or parallel transcription factor networks that cooperate with JAK/STAT signaling.

#### 3.2.3 NF‐κB

IFN‐1 expression has also been found to stimulate NF‐κB via activation of inhibitor of kappa B α (IκBα) phosphorylation and degradation. One study demonstrated that upon IFN‐1 treatment, Daudi cells (Burkitt's lymphoma B lymphoblast cells) displayed an increase in active NF‐κB activity [102]. Further, IFN‐1‐induced NF‐κB activation was found to play a pro‐survival role in cells, indicating a role in tumor promotion during chronic IFN‐1 production. This pro‐survival role is driven by serine phosphorylation and subsequent degradation of the NF‐κB negative regulator, IκBα. In addition to these data, a 2018 study demonstrated that IFN‐1 signaling, through IRF9 and STAT2, cooperates with NF‐κB to promote IL‐6 secretion, promoting immunosuppression and acting as a pro‐tumor mechanism [103]. This indicates that IFN induces classical NF‐κB activation; however, other studies have identified alternative mechanisms of NF‐κB activation [104] as well as a PI3K/AKT dependent activation mechanism [105].

#### 3.2.4 u‐ISGF3

Unphosphorylated ISGF3 (u‐ISFG3) formation and signaling is a more recently discovered member of the noncanonical IFN‐1 stimulated signaling pathway class. The u‐ISGF3 complex can form in the absence of STAT1/2 phosphorylation, and these unphosphorylated complexes are both sufficient and required for some specific IFN‐mediated cellular responses [106, 107]. u‐ISGF3 has been found to exclusively activate the transcription of many genes that mediate prolonged and chronic responses to DNA‐damaging therapies and viral infection. This non‐canonical signaling pathway, further discussed below, is perhaps the least characterized of the non‐canonical mechanisms of action of IFN‐1 mentioned here, warranting further studies into what conditions favor its formation, further defining its signaling, and attempting to better understand its specific phenotypic changes [108].

### 3.3 Hormonal regulation of IFN‐1 signaling

Beyond the implication in regulating IFN‐1 production, biologic sex and hormone signaling may also influence downstream IFN‐1 signaling and outcomes. In female‐associated tissues, steroid hormone signaling pathways intersect extensively with canonical JAK/STAT signaling, potentially altering both the magnitude and functional consequence of IFN‐1 activation.

Interestingly, and relevant to the exploration of female related malignancies, some groups have published on the effects of ER and PR on IFN‐1 (specifically IFN‐ε) and ISG expression. While it is well known that these molecules play key physiologic roles regulating tissues of the FRT and breast, they can also play roles in pathologies such as endometriosis and cancer [109, 110]. Cao et al. found that ERα inhibits IFN‐1 signaling via two mechanisms: activation of ERα both induces the expression of H2A.Z, preventing the ISGF3 complex from binding ISREs in the genome and by inhibiting the formation of ISGF3 complex by interacting with STAT2 to prevent binding of STAT1 and IRF9. These actions ultimately lead to inhibition of ISG expression, a decrease in ‘viral mimicry’, as coined by Sistigu et al., and promote tumorigenesis in a mouse model of breast cancer [62, 111].

PR may play a role in the regulation of ISG expression via inhibition of disrupted ISGF3 formation. One study found that several PR+ breast cancer cell lines were found to have low STAT1 expression and subsequent low ISG expression [112]. A 2017 publication further defined this characteristic and found that STAT1 competes with STAT2 for formation of complexes which stimulate expression of interferon‐responsive genes [113]. Another study found that PR promoted the ubiquitination and degradation of STAT2, further impairing IFN‐1 response [114]. This decrease in STAT1 signaling may allow the tumor to both escape immune surveillance and maintain low‐level, chronic, protumor IFN signaling.

Age‐associated hormonal changes may also influence IFN‐1 signaling dynamics within female‐specific malignancies. Postmenopausal physiology alters IFN‐1 signaling through the combined effects of reduced sex steroid hormones and age‐associated increases in genomic instability, specifically within hormone‐sensitive tissues [115, 116, 117]. The decline in estrogen and progesterone signaling decreases ER/PR‐dependent transcriptional priming of innate immune, TLR7/9, and IRF7 driven programs to lower IFN‐1 responsiveness [118]. Further, long‐term hormonal withdrawal contributes to reduced tissue turnover in estrogen‐responsive tissues, such as the uterus and endometrium, which drives atrophy and increases DNA damage accumulation, chromosome instability, and replication stress [119]. This damage is also associated with an increase in the presence of cytosolic nucleic acids and may contribute to sustained IFN‐1 activation and local inflammation. In hormone‐sensitive malignancies, such as breast or ovarian cancers, this transition may be particularly relevant in that loss of endocrine regulation may weaken immune surveillance but further drive chronic, pro‐tumorigenic IFN‐1 signaling. Thus, menopause may drive shifts in IFN‐1 biology, from a hormonally tuned IFN‐1 program toward a damage‐driven program that can influence both tumor initiation and progression.

These alternative, noncanonical IFN‐1‐stimulated signaling pathways promote tumor specific responses in several cancer types, contributing to female‐associated signaling response plasticity that drives various phenotypes.

### 3.4 Functional outcomes of cancer cell‐autonomous IFN‐1 signaling

#### 3.4.1 Plasticity of an IFN‐1 response

**Fig. 3:**
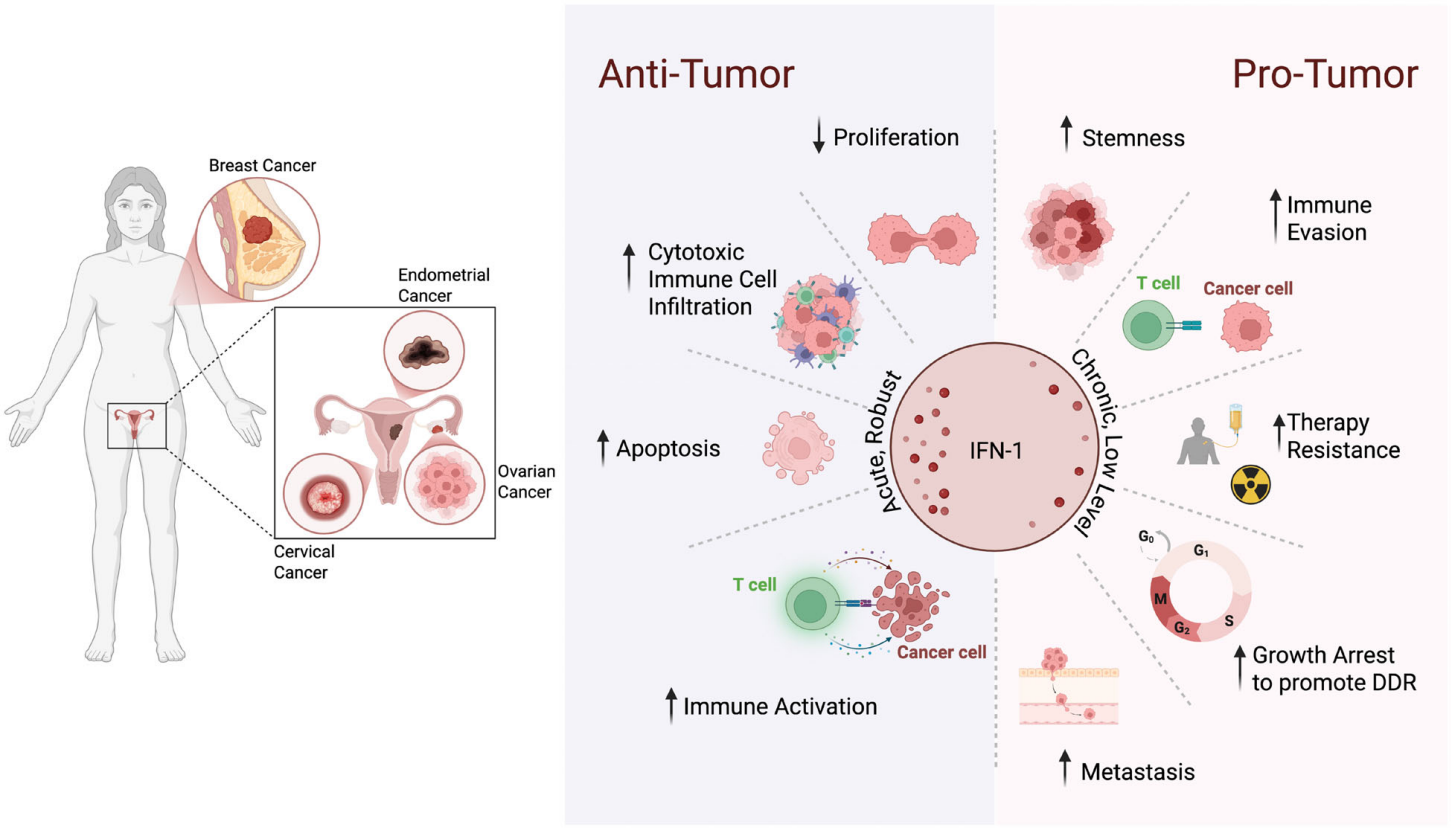
IFN‐1 signaling plasticity in female‐related malignancies. The left image is an icon of an individual from the anterior perspective with callouts to the breast and the female reproductive tract, emphasizing breast cancer, endometrial cancer, cervical cancer, and ovarian cancer. The right graphic indicates various roles of interferon type 1 (IFN‐1) organized based on duration and intensity of the IFN‐1 signal. The left side indicates various antitumor roles of IFN‐1 resulting from acute and robust IFN‐1 signaling and the right side indicates protumor roles resulting from chronic and low‐level IFN‐1. Created in biorender. Conant, A. (2026) https://BioRender.com/9ltaw7t.

The pro‐ vs. antitumor nature of IFN signaling is similar to the ‘classical immune mechanisms of antiviral IFN‐1 response, indicating the role of a tunable IFN response rather than a binary all‐or‐none response [120]. Robust, acute IFN‐1 production and signaling is associated with an antiviral, antitumor response, where antiproliferative genes are activated and ‘tuned’ to the concentration and duration of the IFN‐1 signal [121] (Fig. 3). As demonstrated by Levin et al., the amount of receptor greatly influences the response of this pathway. This group showed that more than 99% of cervical adenocarcinoma cells express a ‘critical threshold’ of IFNAR to allow key antiviral responses to IFN‐1 treatment, while about 60% of cells have a relative receptor threshold to allow an antiproliferative IFN response [122]. These data indicate that the actual amount of receptor, affected by genetic variability, greatly influences this pathway. Several other regulatory mechanisms exist to control IFN‐β induction, such as variation in gene expression of factors that sense viral DNA and affect pathway activation to aid in controlling IFN expression [123].

A cell's ability to make and respond to IFN‐1 is constrained by the stochastic nature of signal transduction cascades, as described in a review by Schrieber et al. [124]. Especially within a nonimmune cell population, the ability of cancer cells to respond to the binding of the IFN‐1 ligand to its receptor, initiating the signaling cascade, is limited to the variability in gene expression of various factors within the pathway. Variability in genetic mutations driving therapy resistance, epithelial‐mesenchymal transition (EMT), stemness, and overall cancer aggression may also lead to IFN‐1 response variability. The signaling and response of cancer cells to interferons is perhaps affected by this random probability of molecular interactions and responses on a cell‐to‐cell basis [123]. Consequently, this signal and response is relatively heterogeneous on a patient‐by‐patient, cancer‐to‐cancer basis. It appears, however, that some generalization can be made regarding the predictability of IFN signaling.

How much and/or how long a cell receives an IFN signal greatly influences the type of response. A study by Jacquelot and colleagues found that prolonged and sustained IFN‐β signaling leads to an upregulation of PD‐L1, contributing to immunotherapy resistance in a mouse mammary carcinoma, colon carcinoma, and fibrosarcoma cell lines [125], suggesting sustained signaling may play a role in pro‐tumor phenotypes. In addition to the effect of IFN‐β on PD‐L1 as demonstrated above, Cheon et al. [126] found that PD‐L1 promotes IFN‐β production and a low‐level, chronic IFN signature which results in resistance to DNA damaging therapies, indicating bi‐directional signaling and a complex feedback loop between PD‐L1, IFN‐β, and pro‐tumor phenotypes. Chronic IFN‐1 signaling, as summarized previously, also promotes the formation and functionality of u‐ISGF3, which is implicated in cyto‐protective functions. These data collectively suggest that the duration and intensity of an IFN‐1 signal is responsible for determining pro‐ vs. antitumor phenotypes (Fig. 3).

To best understand and summarize the functional consequences of this pathway and its plasticity across female‐associated cancers, the studies reviewed here focus on defining the role of cancer cell‐autonomous IFN‐1 signaling, with a particular emphasis on its dynamic and context‐dependent nature.

##### 3.4.1.1 Breast cancer

Historically, three studies generated further interest in the involvement of cancer cell autonomous IFN‐1 production and signaling in therapy resistance and other protumor phenotypes. In 1999, one study utilized microarrays to identify differential gene set expression in human mammary epithelial cells growing in culture, as well as in primary human breast tumors. The authors found that two clusters of genes were correlated with proliferation and IFN‐related signaling. What was not known yet was if this signaling originated specifically from lymphocytes and stromal cells or the cancer cells themselves [127]. Later, in 2004, Khodarev et al. [128] showed that STAT1 was overexpressed in resistant Nu61 tumors when compared to parental, sensitive SCC‐61 tumors and was associated with a tumor‐protective role following radiation therapy. A subset of 52 IFN‐related genes were upregulated following RT, and it was presumed that they played a role in therapy response. The same laboratory later published on the existence of an IFN‐related DNA damage resistance signature (IRDS) that both mediates resistance to DNA damaging therapies and can act as a predictive marker for therapy response in a breast cancer model [57]. The IRDS signature was seen in both breast and ovarian cancer cell lines, although the study was primarily focused on breast cancer. This IFN‐driven signature was found to be exclusively transcribed by the u‐ISGF3 complex, activated following low‐level, prolonged IFN‐β signaling.

A 2006 study comparing breast, prostate, and glioma cells after single and fractionated doses of radiation showed that several IFN‐1‐related genes were robustly upregulated upon multifractionated irradiation in all three tumor types [129]. This group of genes was implicated to play a role in conferring radiation resistance via STAT1 induction. Another study showed, similarly, that an IFN/IRDS signature was present in a patient‐derived xenograft model of breast cancer following chemotherapy treatment. This signature included ISGs and several key IRDS genes. The authors also found that knocking down the IFNAR with an siRNA, while treating cells with mafosfamide, abrogated the effects of CT‐induced IRDS and ISG expression [52]. Interestingly, another group found that high IFNAR1 expression in patients with breast cancer correlates to poor prognosis and decreased progression free survival, as supported on a mechanistic level with an increase in PD‐L1 expression and associated tumor growth in a mouse model [130]. This data indicates that some level of IFN‐1 signaling might promote stemness; however, this role of IFN is not well understood yet. In further addressing the paradigm that the concentration of IFN‐β dictates the response of cancer cells to the signal, Doherty et al. [64] have published that triple negative breast cancer cells expressing very high levels of IFN‐β are significantly less stem cell‐like. These studies may suggest that chronic, low‐level IFN signaling that follows DNA damage promotes pro‐tumor ISG expression, EMT, and a more stem‐like cellular state, while high levels of IFN‐1 decrease overall cancer stemness.

As these phenotypes are highly associated with metastasis, the link between chronic IFN‐1 signaling and breast cancer metastasis remains incompletely understood. A 2012 article details the relationship of IFN‐1 program suppression and metastasis using a preclinical mouse model of triple negative breast cancer. Expression of IRF7, a potent IFN‐α transcriptional activator, and associated interferon pathways were found to be suppressed in isolated bone metastases and rescued with cancer cell‐intrinsic IRF7 induction. Further, overexpression of IRF7 inhibited metastasis and increased survival, indicating a causal link between low‐level IFN‐1 driving pro‐tumor phenotypes and promoting metastasis, while robust IFN‐1 signaling may instead drive antitumor phenotypes favorable for patient outcome [131]. These results strongly support the potential benefit of a STING agonist in enhancing IFN‐1 expression, shifting cells from a pro‐tumor, chronic IFN‐1 signaling state to a robust, cytotoxic IFN response.

BRCA mutations are also associated with ISG activation. Several studies have shown that in breast cancer cells harboring BRCA2 mutations, abundant DNA damage due to defective DDR mechanisms drives a cancer cell intrinsic IFN‐1 response [132, 133]. In one article, cGAS/STING induction following hydroxyurea treatment led to an increase in ISG and IRDS expression which was correlated with a halt in proliferation. This IFN‐1 driven promotion of a non‐proliferative state was hypothesized to allow cells to repair DNA damage. Constitutive IRDS expression causes the cells to switch from cytotoxic antitumor signaling to a prosurvival state, correlating with resistance to DNA damaging therapies in breast cancer [132]. This evidence aids in the understanding of how cancer cell‐intrinsic IRDS and IFN signaling may provide a necessary pro‐tumor survival advantage in cancers treated with CT and/or RT.

Similar to other studies that have demonstrated the induction of IFN‐1 following treatment with DNA damaging agents, Maia et al. [134] found that breast cancer cells secrete IFN‐β following chemotherapy treatment. However, in focusing on the paracrine role of cancer‐intrinsic IFN‐1 production, secreted IFN‐β reprogrammed stromal fibroblasts to an antiviral, interferon‐stimulated state, independent of activation of nucleic acid sensing pathways. This stromal activation promoted cancer cell recovery via cell cycle re‐entry following therapeutic exposure. This study uniquely demonstrates how cancer cell‐intrinsic IFN‐1 production may promote protumor phenotypes through tumor microenvironment reprogramming rather than through cell‐autonomous signaling and response.

Finally, a 2022 study reported that treatment of breast cancer cell lines and tumors with 4‐hydroperoxy‐CPA (an activated cyclophosphamide metabolite) with or without doxorubicin led to increased ISG gene expression and immune cell recruitment, along with notable tumor regression, possibly indicating an antitumor role for IFN‐1 signaling and ISG expression. The effect of 4‐hydroperoxy‐CPA on IFN1 and ISG induction was abrogated with treatment with anti‐IFNAR1, as evidenced by sustained tumor growth indicating that the therapeutic effects of CPA may be dependent on IFN‐1 and associated signaling [135]. These findings are consistent with the work of Sistigu et al. [62], supporting the idea that IFN‐1 response may be necessary for the effectiveness of some therapeutic regimens.

Stepping away from traditional cytotoxic DNA‐damaging therapies, ER+ HER2‐ breast cancer is now primarily treated with cyclin‐dependent kinase 4 and 6 (CDK4/6) inhibitors, which function through targeted suppression of cell cycle progression [136]. Although these agents have significantly improved outcomes in this breast cancer subtype, both intrinsic and developed resistance remain major challenges [137]. De Angelis et al. [138] demonstrated that resistance to CDK4/6 inhibitors *in vitro* is associated with enrichment of IFN‐1 signaling pathways, including expression of the IRDS program along with other ISGs. These findings were supported through *in silico* analyses of publicly available patient data sets, where enrichment of IFN‐1‐associated signatures correlated with poor response to CDK4/6 inhibitors. Among the most predictive genes identified with resistance was STAT1, which was consistently upregulated in samples demonstrating intrinsic CDK inhibitor resistance as well as following both acute and chronic exposure to CDK4/6 inhibition. These findings provide further evidence that chronic tumor cell‐intrinsic IFN‐1 signaling contributes to therapy resistance beyond genotoxic therapies alone and suggest that co‐inhibition of aberrant JAK/STAT activation may be a viable concomitant therapy. This work supports the emerging paradigm that acute and chronic IFN‐1 signaling may play fundamentally distinct roles in cancer biology, with sustained IFN‐1 activation promoting phenotypic plasticity and a resistant state.

##### 3.4.1.2 Ovarian cancer

High‐grade serous ovarian cancer (HGSOC) is the most common and lethal subtype of ovarian cancer and is believed to originate largely from STIC lesions in the fallopian tube. To better define the early molecular events underpinning HGSOC development, Kader et al. [70] performed high‐plex imaging and spatial transcriptomics across stages spanning TP53 mutation, STIC formation, and invasive carcinoma. An IFN and ISG signature—characterized by the enrichment of IFN‐α/γ programs, STAT1, IRDS upregulation, and IFN‐ε downregulation—was detected at all stages, indicating these transcriptional footprints pre‐exist in precancerous lesions and likely contribute to therapy response, immune suppression, and shape immune responses.

Chemotherapy exposure reinforces a near universal IFN‐1 pathway activation in ovarian cancer cells, further indicating the existence and role of a cancer cell‐autonomous signaling program. Wenzel and colleagues [139] demonstrated that isogenic clones, emerging under escalating carboplatin selection, exhibited heightened IFN‐1 signaling and associated slowed proliferation, which was pharmacologically inhibited using the JAK1 inhibitor ruxolitinib. Similarly, a 2023 paper reported on the characterization of a sister pair of sensitive and cisplatin‐induced chemo‐resistant patient‐derived HGSOC cells [140]. The resistant cells were found to be cross‐resistant to both cisplatin and olaparib (a potent poly (ADP‐ribose) polymerase inhibitor), while displaying phenotypic changes that indicated a shift toward a slow‐proliferating, stem‐like phenotype with strong activation of JAK/STAT signaling. A 2026 preprint following these findings observed that direct treatment of both the chemo‐sensitive and ‐resistant cells with low‐level IFN‐β drove cisplatin resistance, and phenocopied chronic cisplatin treatment in several ways [141]. These data suggest IFN‐1 signaling may play a functional role in acquired therapy resistance and associated phenotypes and may represent a targetable program in HGSOC treatment.

Of note, few studies have addressed the direct role of specific IRDS or ISG members in promoting stemness in ovarian cancers. IFI27, both an ISG and IRDS gene, has been shown to promote EMT, stemness, drug resistance, and overall tumorigenicity in ovarian cancer cell lines, suggesting a functional role of IFN‐1 signaling exists in ovarian cancer [63].

Further defining the role of IFN‐1 program activation in HGSOC following therapy, a 2025 preprint performed single‐cell RNA sequencing on seven matched pairs of human ovarian tumors collected pre‐ and postchemotherapy to better identify gene expression changes that play a role in temporary tumor cell chemo‐sensitivity, before regaining resistance to treatment in response to platinum therapy. Winterhoff and colleagues [142] identified an IFN fingerprint, composed of both IFN‐α and IFN‐γ signatures present in 6/7 samples following treatment, and in both cancer cells and stromal cells, indicating that an IFN signature may be a common feature of HGSOC. The authors also noted that ISG+ subpopulations of cells existed within the tumors, without an abundance of immune cells nearby. Of interest, these ISG‐high groups were comprised of predominantly tumor cells, with few neighboring immune cells, indicating the cancer cell‐intrinsic action of the IFN signature in samples following chemotherapy.

Consistent with these models and findings, microarray analysis of platinum‐treated or platinum‐naïve patient‐derived samples, analyzed with respect to platinum sensitivity and proliferation rates, revealed significant inverse correlation between STAT1 and cisplatin sensitivity [143]. To validate this association, STAT1 was overexpressed in a platinum‐sensitive cell line, increasing resistance to cisplatin by three‐ to fivefold, while pharmacologic JAK inhibition significantly sensitized resistant cells. Thus, while these data implicate STAT1 in platinum resistance, the precise contribution of canonical or non‐canonical IFN‐1 signaling remains to be definitively demonstrated.

A later publication found that this STAT1‐mediated resistance was due, in part, to overexpression and interaction of histone deacetylase 4 (HDAC4) and STAT1. Knockdown of HDAC4 validated its role in deacetylation of *STAT1* and resulted in therapeutic sensitization [144]. Interestingly, these authors also established that only resistant cells activate STAT1 in response to cisplatin‐induced DNA damage, while sensitive cells could not mount a similar response. Both phosphorylated and non‐phosphorylated STAT1 were found in the nucleus, in agreement with the idea that low‐level, chronic IFN‐1 drives both ISGF3 and u‐ISGF3 signaling, with the u‐ISGF3 complex being exclusively responsible for transcription of the IRDS gene set.

BRCA2 mutant cells were also found to promote a late, chronic upregulation of IFN‐1‐related response genes. This response is seen alongside robust cGAS/STING/STAT1 pathway activation, suggesting a role for DDR deficiency in driving a protumor IFN‐1 signal and response in ovarian cancer specifically [81].

Together, these findings indicate that genomic instability and DNA damage converge to promote chronic IFN‐1 signaling‐driven pro‐tumor phenotypes in HSGOC.

##### 3.4.1.3 Cervical cancer

Since HPV is the most common etiological factor for cervical cancers, it is difficult to discern if IFN‐1 signaling in this context reflects a robust, sustained antiviral response or the chronic low‐level response as discussed above. One study found that IFN‐α induced apoptosis in cervical cancer HeLa cells via both the intrinsic and extrinsic apoptotic pathways, indicating a potent antiviral role of the cytokine [145]. Another article found that Bcl2‐associated athanogene 2 (BAG2) inhibited the progression of cervical cancer by stabilizing STING overexpression, promoting IFN‐1 production, and increasing ISG expression [146]. STING activation and this IFN‐1 induction in this context is likely driven by cGAS detection of cytosolic DNA from DNA damage, micronuclei, or viral genomes. These articles indicate that a robust antiviral IFN response is beneficial in this cancer type, as it inhibits cervical cancer tumor progression.

##### 3.4.1.4 Endometrial cancer

Within endometrial cancer, POLE exonuclease domain mutations are both common and clinically significant, forming a distinct subtype (POLE‐mut) in modern classification systems [147]. POLE mutations drive DNA damage by impairing leading‐strand proofreading during DNA replication, driving ultra‐mutation, replication stress, and accumulation of DNA damage that can generate cytosolic DNA and drive activation of the cGAS/STING/IFN‐1 axis. Notably, the recurrent POLE P286R mutation has been associated with an increase in total cGAS, STING, and IFN‐β protein found in tumors derived from humanized mouse xenograft models of endometrial cancer [148]. The same study demonstrated upregulation of IRDS gene members, IFIT1 and IFIT3 (interferon‐induced protein with tetratricopeptide repeats 1/3), on POLE P268R‐mutant endometrial cancer cells, supporting a functional link between replication‐associated genomic instability and tumor‐cell intrinsic IFN‐1 signaling program activation.

Similarly, MSI‐H (microsatellite‐high) endometrial tumors, which arise from mismatch repair deficiency and consequent insertion–deletion mutations within microsatellite regions, display marked genomic instability that can enhance innate immune signaling pathways [149]. Across several endometrial cancer specific molecular subtypes, increased STAT1 activation, elevated ISG expression, and high PD‐L1 expression have been observed and potentially contribute to adaptive therapy resistance and tumor progression [150, 151, 152]. Of note, DNA methylation‐driven expression of an IRDS gene, ISG15, has been associated with poor outcomes and late staging in several subtypes of endometrial cancer, with serous endometrial carcinoma displaying the highest levels of ISG15 expression and endometroid endometrial carcinoma with the lowest [153]. This gene was also associated with high expression of PD‐L1, further indicating its potential role in promoting an antitumor phenotype and contributing to disease progression. While these data provide little knowledge of IFN signaling driving the ISG expression, it details the role of ISG activation in the progression of the malignancy.

Although these molecular subtypes are strongly associated with tumor progression and distinct IFN‐related program activation, further studies are required to define the extent to which specific subtypes and genetic markers, such as POLE and MSI‐H, are associated with IFN‐1 signaling functions in a cell autonomous manner to promote or inhibit endometrial tumor progression or aggression. Thus, the precise role of IFN and related signaling in endometrial cancer has yet to be fully understood and seems to remain paradoxical.

## 4 Therapeutic potential in female‐related malignancies

Despite early enthusiasm over the potential use of IFN in cancer therapeutics, in the late 1990s and early 2000s, clinical results of exogenous IFN treatment disappointed clinicians and researchers. In fact, these results were so disappointing that IFN developed a clinical reputation as a ‘dead drug’ [154]. Within the past several years, as preclinical research has led to a better understanding of the biochemical behavior and function of these cytokines, over 100 clinical trials involving treatment with exogenous IFN, agonists and antagonists of IFN production and signaling, both alone and in conjunction with other therapies, have now been completed. Many of these trials showed positive results, showcasing the promise of this new frontier of cancer immunotherapy (Table 2).

**Table 2:** Summary of clinical and pre‐clinical trial landscape utilizing IFN‐1 agonizing and antagonizing therapeutics.

Agent	Class	Target	Tumor type	Trial phase	ClinicalTrials.gov ID	Outcome(s) studied	Status
E7766	STING agonist	Intratumoral Injection	Agnostic; advanced unresectable solid tumors	Phase 1 Clinical	NCT04144140	*Primary*: Maximum tolerated dose and/or recommended phase 2 dose *Secondary*: safety/tolerability profile	Published
Manganese chloride (MnCl2) in combination with nab‐paclitaxel, cisplatin, sintilimab	STING agonist	Inhalation, Intravenous infusion	Platinum resistant or refractory ovarian cancer	Phase 2 randomized open label	NCT03989336	*Primary*: Objective response rate, safety *Secondary*: disease control rate, progression‐free survival, oberall survival	Completed, presented ASCO 2020
Ulevostinag (monotherapy or combination with pembrolizumab)	STING agonist	Intratumoral injection	Advanced/metastatic solid tumors, lymphoma (expansion phase: head and neck squamous cell carcinoma, triple negative breast cancer)	Phase 1	NCT03010176	*Primary*: Safety, tolerability, identifying recommended phase II dose *Exploratory*: biomarkers	Published
Ulevostinag (monotherapy or combination with pembrolizumab)	STING agonist	Intratumoral injection	Metastatic or unresectable, recurrent head and neck squamous cell carcinoma	Phase 2	NCT04220866	*Primary*: antitumor activity	Published
BI 1987446 (monotherapy or combination with ezabenlimab)	STING agonist	Intravenous infusion	Advanced, metastatic, unresectable, relapsed/ refractory solid tumors	Phase 1	NCT05471856	*Primary*: Safety of escalating doses, maximum tolerated dose *Secondary*: efficacy, safety, pharmacokinetics, and pharmacodynamics	Recruiting
CKM (rintatolimod, IFN‐⍺2b, celecoxib) with paclitaxel, dose‐dense doxorubicin and cyclophosphamide, surgery	IFN replacement	Intravenous infusion	Triple negative breast cancer	Phase 1	NCT04081389	*Primary*: Safety *Secondary*: efficacy	Published
Pegintron (combination with p53 vaccine and gemcitabine)	IFN replacement	Intramuscular injection	Platinum resistant ovarian cancer	Phase 1/2	NCT01639885	*Primary*: Feasibility and immunogenicity of the combination regimen *Secondary*: immune system impact, relationship between antitumor immunity and cinical outcome	Published
VSV‐hIFNbeta‐NIS (monotherapy or combination with ruxolitinib)	IFN replacement	Intravenous infusion	Stage IV or recurrent endometrial cancer	Phase 1	NCT03120624	*Primary*: Determine optimal dose schedle, safety/tolerability *Secondary*: toxicity profile, viral outcomes, humoral response, tumor response rate, overall survival	Active, not recruiting
H‐151 (combination with cisplatin)	STING antagonist	Cell culture, Allograft mouse model intraperitoneal injection	Ovarian Cancer Cell Lines	Preclinical		*Primary*: Determine correlation between STING expression levels and the response to platinum treatment in ovarian cancer	Published
5‐azacytidine (combination with DNA methyltransferase and histone deacetylase inhibitors)	IFN agonist	Intraperitoneal treatment	Ovarian Cancer Murine Model	Preclinical		*Primary*: Quantify reversal of tumor‐microenvironment immune‐evasion	Published
Ruxolitinib (monotherapy)	JAK1/2 inhibitor	Oral	Metastatic triple negative breast cancer	Phase 2	NCT01562873	*Primary*: Objective response rate *Secondary*: progression‐free survival, overall survival, toxicity profile, clinical benefit rate	Published
Ruxolitinib (combination with neoadjuvant carboplatin, dose‐dense paclitaxel)	JAK1/2 inhibitor	Oral	Newly diagnosed advanced ovarian, fallopian tube, or primary peritoneal cancer	Phase 1/2	NCT02713386	*Primary*: Progression‐free survival *Secondary*: frequency/severity of adverse events, overall survival, rates of total gross resection, rates of complete pathologic response	Published
Ruxolitinib (combination with pembrolizumab)	JAK1/2 inhibitor	Oral	Metastatic triple negative breast cancer	Phase 1	NCT03012230	*Primary*: Maximum tolerated dose *Secondary*: Treatment safety and efficacy	Completed, presented SABCS 2023

### 4.1 Activating IFN production and signaling

As discussed above, chronic, low‐level IFN‐1 production appears to be protumorigenic in many models, while robust, high‐level IFN‐1 is cytotoxic; thus, identifying therapies capable of enhancing IFN‐1 signaling to shift the balance toward antitumor activity remains an important area of investigation. Therapeutic approaches that amplify IFN‐1 signaling have shown promise in female malignancies. The dichotomous function of this cytokine and its related pathways must be better understood in specific patient populations before a more generalized therapeutic approach to increasing low‐level, protumor IFN production can be taken. Several clinical trials, reviewed below, have studied the effects of recombinant interferon therapies or STING agonists to activate the transcription of the cytokine, in order to treat various female‐related malignancies [155, 156, 157].

In attempting to determine how best to promote IFN production and signaling in malignancies where low‐level IFN‐1 production and signaling drives pro‐tumor effects, various therapeutic approaches have been explored. The most promising of these include the use of STING agonists to stimulate IFN‐1 and promote related signaling pathways, as well as the administration of exogenous recombinant IFN‐1 in combination with other conventional cancer therapeutics [158, 159, 160].

In a tumor agnostic phase 1 clinical trial (NCT04144140) of 24 patients with advanced, unresectable solid malignancies, intratumoral injection of a novel STING agonist, E7766 resulted in increased plasma levels of IFN‐α, IFN‐β, and IFN‐γ, as well as additional proinflammatory cytokines, within 10 h of injection, before they dropped to near‐baseline levels around 24 h postinjection, indicating a robust response to STING stimulation. STING agonist therapy resulted in an increase in RNA and protein expression levels of PD‐L1, percentage of CD8+ lymphocytes, and STING gene expression post treatment. The authors noted that neither pharmacodynamic nor clinical response was dose dependent, with stable disease seen in two patients receiving the lowest dose, tumor regression in non‐injected lesions in another patient, and increased levels of CD8+ effector T cells at low treatment doses, compared to increased levels of pro‐inflammatory cytokines at higher treatment doses. The heterogeneous patient population and resulting heterogeneity among responses without a dose‐dependent correlation highlight the challenges in elucidating optimal dosing to attain a balance between activation of appropriate immune response while limiting excessive inflammation. Notably, a consistent decrease in overall tumor size was not observed, and tumor growth was seen in the majority of patients, indicating the need for further studies among a more homogeneous patient population, which may allow for better generalization of therapeutic dosing [156].

Similarly, in a 2025 phase II randomized, open‐label study (NCT03989336), Zhang et al. [157] utilized manganese chloride (MnCl2), an inhalable STING agonist, in combination with nab‐paclitaxel, cisplatin, and sintilimab, a PD‐1 inhibitor, in 27 heavily pretreated (median prior therapy lines = 4) patients with ovarian cancer. Preliminary results demonstrated a 78.6% objective clinical response rate and disease control was achieved in all patients receiving MnCl2 (11 partial responses, 3 stable diseases). Encouragingly, partial response was seen by first interim evaluation in 71.4% of patients, and the addition of STING agonism did not significantly exacerbate treatment‐related adverse events.

A recent 2025 publication reported on the safety and tolerability of intratumoral delivery of STING agonist ulevostinag, both as a monotherapy and in combination with pembrolizumab, in head and neck squamous cell carcinoma and triple negative breast cancer (NCT03010176, NCT04220866). While not explicitly stated, this study supports a key concept discussed in this review: that cancer cell–intrinsic STING activation in tumors with low baseline IFN production and signaling may promote antitumor signaling and immune cell recruitment. Using an accelerated titration design with a modified toxicity probability interval method, the authors reported moderate toxicity of the agonist, the most common adverse effect being a grade 3–4 fever (observed in 70% of patients). Although no objective responses were observed in patients with advanced or metastatic disease, dose‐dependent pharmacokinetics were observed, showing increases in STING‐related RNA expression signatures and circulating CXCL10—peaking 6–8 h post‐treatment—indicating robust STING pathway activation and immune stimulation. When used in combination with pembrolizumab, ulevostinag demonstrated a 4% objective response rate in breast cancer patients, compared to 6–31% response rates reported for other second‐line therapies [160, 161]. Although this study observed some antitumor ability of ulevostinag when used intratumorally, further studies of ulevostinag have been discontinued due to the challenges of the delivery method when compared to the successes seen using similar agonists via systemic administration alongside other chemo‐ and immunotherapies.

A current phase 1a open label dose‐escalation clinical trial (NCT05471856) seeks to study the effects of a novel STING agonist, BI 1987446, in combination with ezabenlimab (PD‐L1 antagonist) in patients with advanced solid tumors. This study aimed to overcome the resistance commonly seen with immune checkpoint inhibitor therapy through dual pathway immunostimulation. This study is actively recruiting and has not yet provided preliminary results [155].

### 4.2 IFN‐1 replacement

The Roswell Park Comprehensive Cancer Center has completed a phase 1 interventional study (NCT04081389) focused on the combination of systemic chemokines in conjunction with neoadjuvant chemotherapy in the treatment of early‐stage triple negative breast cancer. The treatment, termed CKM, consisted of a selective TLR3 agonist (*rintatolimod*), interferon (*IFN‐α2b*) administered in three escalating doses, and cyclooxygenase‐2 (COX‐2) inhibitor (celecoxib). CKM was administered in conjunction with paclitaxel for 3 weeks, followed by 9 weeks of paclitaxel alone, dose‐dense doxorubicin and cyclophosphamide, and surgery. The combination treatment was well‐tolerated, with no dose‐limiting toxicities observed. At the time of surgery, 56% of patients had complete pathologic response, and 10% of patients had only microscopic residual disease. These results are comparable to those seen when patients receive neoadjuvant chemotherapy with pembrolizumab. The authors also found that CKM/paclitaxel combination resulted in an inflammatory response in the tumor as well as in the blood to the TME. CXCL12, a key protein in shaping the tumor immune microenvironment, was also found in the TME and blood of patients receiving CKM/paclitaxel, indicating that this treatment regimen may activate pathways that dampen immune responses [162]. A phase II trial is currently planned to confirm the efficacy of the CKM/neoadjuvant chemotherapy regimen.

Similarly, a 2015 clinical trial (NCT01639885) studied the combination of exogenous IFN‐1, p53 vaccine, and gemcitabine in 18 patients with p53 mutated platinum resistant ovarian cancer [163]. Three patients received gemcitabine alone, nine received gemcitabine + Pegintron (IFN‐*α*), and six received gemcitabine + Pegintron + p53 SLP (synthetic long peptide vaccine). Fourteen patients did not complete the planned treatment course due to disease progression. 50% of patients in the study experienced grade 3/4 adverse events, most commonly nausea/vomiting (22%) and dyspnea (17%), and four patients required hospital admission due to the severity of nausea/vomiting. The majority of patients experienced fatigue (78%), flu‐like symptoms (72%), and nausea/vomiting (67%), most of which were grade 1–2. Patients receiving the p53 SLP vaccine universally developed grade 1–2 local skin reactions, 82% of which persisted 2months after treatment. Consistent with the study's primary objective to determine the immunogenicity of the regimen, treatment induced a profound activation of CD4+ and CD8+ T‐cells, although not regulatory, contributing to general immune stimulatory effects. CT assessment demonstrated partial response in two patients and stable disease in four; however, the study was not designed to detect clinical benefit. Future studies exploring IFN‐1 in combination with standard treatments will be needed to further elucidate the role of IFN in both immune activation and cancer cell‐intrinsic signaling.

Another study utilized a vesicular stomatitis virus‐human interferon beta‐sodium iodide symporter (VSV‐hIFNbeta‐NIS) with or without ruxolitinib phosphate (to inhibit JAK/STAT signaling) to treat patients with recurrent or stage IV endometrial cancer (NCT03120624). The purpose of this unique study design is to deliver high levels of IFN‐β directly to tumor cells (tracked with the presence of iodine, resulting from viral expression of the sodium iodide symporter (NIS)), thus driving cytotoxic effects. The group also aims to use ruxolitinib to prevent cell growth and proliferation pathway activation, with the hypothesis that giving both the virus and ruxolitinib may be more effective at treating stage IV endometrial cancer than the virus alone. There are no publications or updates from the study yet; however, the main goal of the study is to determine optimal dose, dose schedules, and safety/toxicity profiling of both the virus and ruxolitinib.

These early phase studies typically focus on increasing IFN‐1 levels, with the primary objective of understanding the resulting immune response, as opposed to assessing clinical response. Further studies correlating pretreatment levels of IFN‐1 with treatment‐induced immunologic changes and clinical tumor response are warranted to elucidate which patients might benefit from such treatments.

### 4.3 Blocking IFN production and signaling

Developing successful IFN‐based treatment regimens addressing cancer cell‐intrinsic IFN production and signaling relies on the ability to characterize tumor IFN and ISG activity. In cases where chronic IFN production promotes therapy resistance, blocking IFN signaling and ISG expression, especially concurrently in combination with a traditional DNA damaging agent, may be a successful mechanism of treatment. Targeting upstream regulators of this pathway, such as cGAS/STING, may help restore transient, robust, antitumorigenic IFN signaling, while blocking the signaling cascade using an anti‐IFNAR1 antibody or JAK inhibitor may help mediate the downstream pro‐tumor phenotypic changes.

There are few studies utilizing STING antagonists; however, one preclinical study utilized cell lines and patient samples to characterize cisplatin treatment, therapy resistance, STING activation, and IFN promotion in cancer‐associated fibroblasts (CAF). The activation of this pathway in CAFs drove cisplatin resistance in ovarian cancer cells, likely due to chronic IFNβ signaling. This study used a STING antagonist, H‐151, to reverse these effects of cisplatin‐induced chronic IFNβ signaling. Using a patient derived ovarian cancer xenograft model, H‐151 was administered in combination with cisplatin, resulting in significant tumor size reduction when compared to treatment with cisplatin alone [164]. Similarly, Stone et al. [165] conducted an *in‐vivo* murine ovarian cancer study to determine if the immune suppressive nature of ovarian cancer can be reversed by blocking IFNAR1 while treating with 5‐azacytidine (AZA) and histone deacetylase inhibitors (HDACi). The results indicated that the epigenetic drug drives significant increases in CD8+ T cell and NK cell activation in the tumor microenvironment, correlating with a decrease in tumor burden and an increase in overall survival of mice. α‐IFNAR1 abrogated the effects of epigenetic modifiers, specifically AZA, indicating that the antitumor action of AZA is mediated by IFN‐1. Blocking IFN thus prevents robust immune activation seen with the treatments listed above. These data indicate that the combination of an HDACi alongside AZA might be the key to reversing the immuno‐evasion phenotype of ovarian cancer, thus sensitizing cells to checkpoint inhibitors.

Ruxolitinib is an FDA‐approved selective JAK1/2 inhibitor, currently approved for use in intermediate or high‐risk myelofibrosis and polycythemia vera (PV). Several groups are exploring the use of JAK1/2 blockade in certain cancers, given that overactive JAK/STAT signaling drives inappropriate cellular proliferation, tumorigenic immune signaling, and treatment resistance. A previous clinical trial (NCT01562873) has shown that Ruxolitinib is generally not effective as a single‐agent therapeutic [166]; thus, the current focus is evaluating the use of this drug in combination with standard‐of‐care treatments.

The NRG Oncology Study group completed a phase 1/2 clinical trial (NCT02713386) of the combination of ruxolitinib, carboplatin, and dose‐dense paclitaxel in patients with ovarian, fallopian tube, or primary peritoneal carcinoma undergoing neoadjuvant chemotherapy in the upfront setting [167]. The study regimen (ruxolitinib 15 mg orally twice‐daily, in conjunction with neoadjuvant chemotherapy) was generally well‐tolerated, with a trend toward higher rates of grade 3/4 hematologic and thrombotic toxicities. Median progression‐free survival (PFS) increased by 3 months in the experimental arm (11.6 vs. 14.6 months, hazard ratio 0.702, log‐rank *P* = 0.059). Further studies of this treatment regimen are warranted; however, the results must be interpreted with caution, as dose‐dense paclitaxel is no longer the standard of care in the neo‐adjuvant setting. Given the role of the JAK/STAT pathway in the development of chemoresistance, use of ruxolitinib may be particularly warranted in the maintenance phase to prevent the regrowth of the residual chemo‐resistant cancer population.

Finally, an ongoing phase I trial (NCT03012230) aims to determine the maximum tolerated dose (MTD), safety, and efficacy of ruxolitinib in combination with pembrolizumab in the treatment of chemotherapy refractory stage IV triple negative breast cancer. Although no results have been published yet, a 2023 abstract indicated that the combination therapy was well tolerated at all doses, although there were more adverse events noted at higher doses of ruxolitinib. No patients had clinical disease response; however, two out of six patients treated with dose level 0 or 1 ruxolitinib had stable disease for 6 months, suggesting that the combination treatment may have more activity when administered with pembrolizumab [168].

Of note, Anifrolumab, a human monoclonal antibody targeting IFNAR1, received FDA approval in 2021 for the treatment of systemic lupus erythematosus. Despite its success in the autoimmune setting, there are currently no active clinical trials investigating the use of IFNAR1 inhibition in female malignancies.

The continued research of the cGAS/STING and JAK/STAT pathways in relation to varying levels of ISGs and varying ranges of cancer cell phenotypes has been proven to be integral to the progression of therapy for a wide range of female malignancies. Of interest, the majority of these studies are concerned with the immunologic effects of these drugs, rather than the cancer cell‐intrinsic effects in relation to the clinical treatment response. The dual nature of the STING signaling pathway, IFN‐1 itself, and JAK/STAT results in a wide range of therapeutic approaches that warrant further exploration, given the various successes seen in the past few years. Many of these studies use activation of the immune compartment as a measure of success. Studies observing the effect of drugs such as the ones listed above will facilitate a better understanding of the dualistic role of cancer cell autonomous IFN production and signaling in various cancers.

## 5 Conclusion

Underlying mechanisms initiating chronic IFN‐1 production and signaling in cancer remain poorly understood. Evidence across female‐related malignancies suggests that the magnitude, duration, and context of IFN‐1 signaling shaped by tumor genotypes, therapeutic stress, and microenvironmental conditions may drive pro‐ or antitumor phenotypes. In ovarian cancer in particular, studies have identified evidence of functional IFN‐1 program activation as early as p53 mutation in premalignant lesions. These observations highlight an important opportunity to leverage IFN and ISG expression patterns as potential candidates of predictive biomarkers of therapy response and recurrence risk within the ovarian cancer setting. However, the phenotypic plasticity associated with IFN signaling, including its ability to promote cytotoxic responses in some contexts while driving senescence, stemness, and therapy resistance in others remains highly understudied. A deeper mechanistic understanding of the dualistic role of IFN and its related divergent outcomes will be essential for determining when therapeutic modulation of IFN‐1 signaling is beneficial, thus eliciting a call to action for researchers and clinicians to identify how this pathway can be exploited to improve patient treatment outcomes.

Importantly, dysregulated IFN programs may present actionable therapeutic vulnerabilities in female‐related cancers, either using off‐label therapeutic regimens or through the discovery of novel therapeutics. Theories of sex‐biased immune regulation suggest that XX individuals may mount stronger IFN‐1 responses due to the combined effects of estrogen‐mediated enhancement of the signaling pathway and increased dose of X‐linked immune genes that escape X‐chromosome inactivation. These factors, including hormonal influence and X chromosome dosage, have been associated with heightened IFN responsiveness, which promotes more robust innate immune activation and may represent a therapeutically actionable feature in female‐associated cancers, where IFN‐1 signaling often presents as pro‐tumor.

In breast, endometrial, and ovarian tumors exhibiting chronic, protumor IFN activation driven by tumor specific mutations or genetic instability, strategies targeting upstream or downstream IFN‐1 signaling molecules may be beneficial. cGAS/STING inhibitors may aid in the modulation of DNA damage immune activation responses, while JAK/STAT or ISG inhibitors may help suppress protumor phenotypic changes associated with a sustained IFN‐1 signal. Emerging evidence suggests that unique interferons, such as IFN‐ε, which are hormonally regulated in the FRT, could represent a unique therapeutic axis for modulating local IFN‐1 activation without inducing systemic toxicity typical of other IFN‐1‐based therapies. Similarly, targeting defining mutations of these malignancies, which have been associated with sustained IFN‐1 activation (POLE, ER/PR, BRCA1/2), may aid in treatment by influencing tumor progression and therapeutic responses.

Finally, improved clinical monitoring of IFN‐1 pathway activation and dynamics may enable more precise patient stratification and subtyping. Liquid biopsy approaches such as profiling circulating tumor DNA, extracellular vesicles, and circulating tumor cells could provide minimally invasive and clinically feasible methods to track IFN‐1‐associated program activation before and during treatment. These approaches may help identify patients transitioning toward therapy‐resistant IFN‐1 states and guide adaptive therapeutic innovation. These directions emphasize the importance of integrating sex‐specific biology, IFN‐1 signaling, and tumor evolution to develop more effective and equitable treatment strategies for women with cancer. Thus, conversations such as this provide knowledge required for sex‐specific medicine and equitable therapeutic strategies.

## Conflict of interest

The authors declare no conflict of interest.

## Author contributions

AC and JJU were involved in conceptualization. JJU and YJI were involved in funding acquisition. AC, KM, and NB were involved in visualization. AC, NB, SA, and JJU were involved in writing—original draft. AC, NB, SA, KM, VS, TS, YJI, and JJU were involved in writing—review and editing.

## Data Availability

Data sharing not applicable; no new data were generated for this review.
